# Physiology-Enhanced Data Analytics to Evaluate the Effect of Altitude on Intraocular Pressure and Ocular Hemodynamics

**DOI:** 10.3390/photonics9030158

**Published:** 2022-03-05

**Authors:** Alice Verticchio Vercellin, Alon Harris, Aditya Belamkar, Ryan Zukerman, Lucia Carichino, Marcela Szopos, Brent Siesky, Luciano Quaranta, Carlo Bruttini, Francesco Oddone, Ivano Riva, Giovanna Guidoboni

**Affiliations:** 1 Department of Ophthalmology, Icahn School of Medicine at Mt. Sinai, New York, NY 10029, USA; 2 Department of Ophthalmology, Indiana University School of Medicine, Indianapolis, IN 46202, USA; 3 Department of Ophthalmology, Miller School of Medicine, University of Miami, Miami, FL 33136, USA; 4 School of Mathematical Sciences, Rochester Institute of Technology, Rochester, NY 14623, USA; 5 MAP5 UMR CNRS 8145, Université de Paris, 75006 Paris, France; 6 Department of Ophthalmology, Centro Oculistico Italiano, 25122 Brescia, Italy; 7 Department of Surgical & Clinical, Diagnostic and Pediatric Sciences, Section of Ophthalmology, University of Pavia—IRCCS Fondazione Policlinico San Matteo, 27100 Pavia, Italy; 8 Glaucoma Unit, IRCCS—Fondazione Bietti, 00198 Rome, Italy; 9 Department of Ophthalmology, Istituto Clinico Sant’Anna, 25127 Brescia, Italy; 10 Department of Electrical Engineering and Computer Science, University of Missouri, Columbia, MO 65211, USA; 11 Department of Mathematics, University of Missouri, Columbia, MO 65211, USA

**Keywords:** glaucoma, altitude, intraocular pressure, mathematical modeling, physiology-enhanced data analytics

## Abstract

Altitude affects intraocular pressure (IOP); however, the underlying mechanisms involved and its relationship with ocular hemodynamics remain unknown. Herein, a validated mathematical modeling approach was used for a physiology-enhanced (*pe-*) analysis of the Mont Blanc study (MBS), estimating the effects of altitude on IOP, blood pressure (BP), and retinal hemodynamics. In the MBS, IOP and BP were measured in 33 healthy volunteers at 77 and 3466 m above sea level. *Pe-retinal hemodynamics* analysis predicted a statistically significant increase (*p* < 0.001) in the model predicted blood flow and pressure within the retinal vasculature following increases in systemic BP with altitude measured in the MBS. Decreased IOP with altitude led to a non-monotonic behavior of the model predicted retinal vascular resistances, with significant decreases in the resistance of the central retinal artery (*p* < 0.001) and retinal venules (*p* = 0.003) and a non-significant increase in the resistance in the central retinal vein (*p* = 0.253). *Pe-aqueous humor* analysis showed that a decrease in osmotic pressure difference (OPD) may underlie the difference in IOP measured at different altitudes in the MBS. Our analysis suggests that venules bear the significant portion of the IOP pressure load within the ocular vasculature, and that OPD plays an important role in regulating IOP with changes in altitude.

## 1. Introduction

Glaucoma is characterized by the loss of retinal ganglion cells and changes to the optic nerve head that are associated with progressive irreversible vision loss. Glaucoma is the second leading cause of blindness worldwide, and by 2040, it is estimated to afflict 111.8 million persons [1]. Currently, intraocular pressure (IOP) is the only modifiable risk factor for glaucoma, with research having established IOP reduction as effective in delaying or preventing the onset and progression of the disease [2]. Other, currently less-modifiable risk factors have been identified in the disease pathogenesis including deficiencies in both systemic and ocular hemodynamics [3–5]. Importantly, these risk factors are known to be influenced by altitude; however, the underlying mechanisms and the clinical significance of altitude’s effects on them have not been established.

During increasing elevation, oxygen saturation within the blood decreases due to the depressed atmospheric pressure [6,7]. This results in a cascade of systemic responses, including an increase in resting blood pressure and heart rate and autoregulatory alterations to cerebral blood flow secondary to hypoxia [8–11]. In comparison, the literature regarding altitude-related alterations in IOP and ocular hemodynamics is much less clear. While several studies have indicated IOP may increase with altitude [6,12–22], many have also found an opposing decrease in IOP with altitude [7,8,23–32]. Additionally, studies have shown that short-term exposure to high altitudes can alter the retinal microcirculation in young, healthy subjects secondary to hypoxic endothelial dysfunction [33]. Several studies have also noted ocular vascular changes with ascension including retinal vessel dilation and alterations in retinal and choroidal blood velocity and pressure [8,10,21,33,34]. However, these effects are generally self-limiting and nonindicative of lasting ocular or systemic hemodynamic changes [35–37].

The mechanisms involved and the consequences of altitude-related changes to IOP and ocular hemodynamics are important to understand for mitigation of disease risk, including glaucoma. Mathematical modeling techniques allow for the creation of a virtual laboratory for the testing of hypotheses in a controlled environment, allowing researchers to better understand the inputs, proposed mechanisms, and outcomes of modeled experiments [38]. In this perspective, herein, we combined experimental measurements with mathematical modeling to better understand the relationships between altitude, IOP, and hemodynamic changes and the clinical significance of these alterations on ocular disease. Specifically, the data previously collected from the Mont Blanc Study (MBS) [32] were used as inputs for two mathematical models describing the physiology of ocular hemodynamics [39] and aqueous humor flow [40]. The outputs of these models provide estimates for variables that bear physiological interest but that cannot be measured directly. Thus, the methodological approach proposed in this study, henceforth referred to as *physiology-enhanced data analytics*, will model and quantify the physiologic relationships between changes in IOP, blood pressure (BP), and ocular hemodynamics. Outcomes from the model will provide a framework for future altitude studies and provide needed clarity on the influence of altitude on IOP, BP, ocular hemodynamics, and associated risks for ocular pathologies.

## 2. Materials and Methods

### 2.1. The Mont Blanc Study

The MBS measured IOP, central corneal thickness (CCT), systolic BP (SBP), diastolic BP (DBP), heart rate (HR), and oxygen saturation in 33 healthy volunteers (17 females, 16 males; mean age: 24.8 ± 3.3) at 77 m (Pavia, Italy (PV)), at 1300 m (Courmayeur, Italy (CM)), and at 3466m (Pointe Helbronner, Mont Blanc Mountain, Italy (PH)) above sea level [32]. The ascent from CM was performed via a high-speed cableway, which took 15 min to reach PH (Skyway Mont Blanc cable car, Skyway Monte Bianco, Funivie Monte Bianco S.p.a, Aosta Valley, Italy). IOP was measured by applanation tonometry (Perkins MK2, Clement and Clarke) and rebound tonometry (I care TAO1i tonometer, Icare Finland Oy), under topical anesthesia with Oxybuprocaine drops. CCT was assessed by ultrasound pachymetry (PachMATE2). Subjects were examined at 9 a.m., 11 a.m., 1 p.m., and 3 p.m. in an indoor setting at PV and PH. IOP and CCT were also measured outdoors at PH at 9 a.m. [32]. Subjects were not acclimatized, nor were they required to exert any physical effort. One eye was selected for study throughout its course.

The study found mean values of IOP to significantly decrease from PV to PH with multiple tonometers [32]. Additionally, CCT was found to increase with ascension, largely matching the current literature [12,13,19,25,32]. The study initially hypothesized that hypoxic conditions found at higher altitudes may deplete the oxygen supply to nonpigmented ciliary epithelial cells, reducing the production of aqueous humor and thus IOP [32]. The MBS provides an essential experimental framework for this study to better understand the physiologic association between IOP, BP, ocular hemodynamics, and altitude.

### 2.2. Mathematical Model of Retinal Hemodynamics

The mathematical model proposed and validated in [39] to study the hemodynamics in the retina was used in combination with the data from the MBS to assess how changes in BP and IOP due to different altitudes affect retinal hemodynamics. In the model, arterial BP is the driving force of the blood flow through the vasculature, which includes the central retinal artery and vein (CRA and CRV) and the retinal microvasculature (retinal arterioles, capillaries, and venules), whereas IOP acts as an external pressure on the vessels. In this study, the model was used in a novel way to obtain individualized estimates of hemodynamic variables based on individualized pressure inputs, thereby enabling physiology-enhanced data analytics.

**Figure 1.**
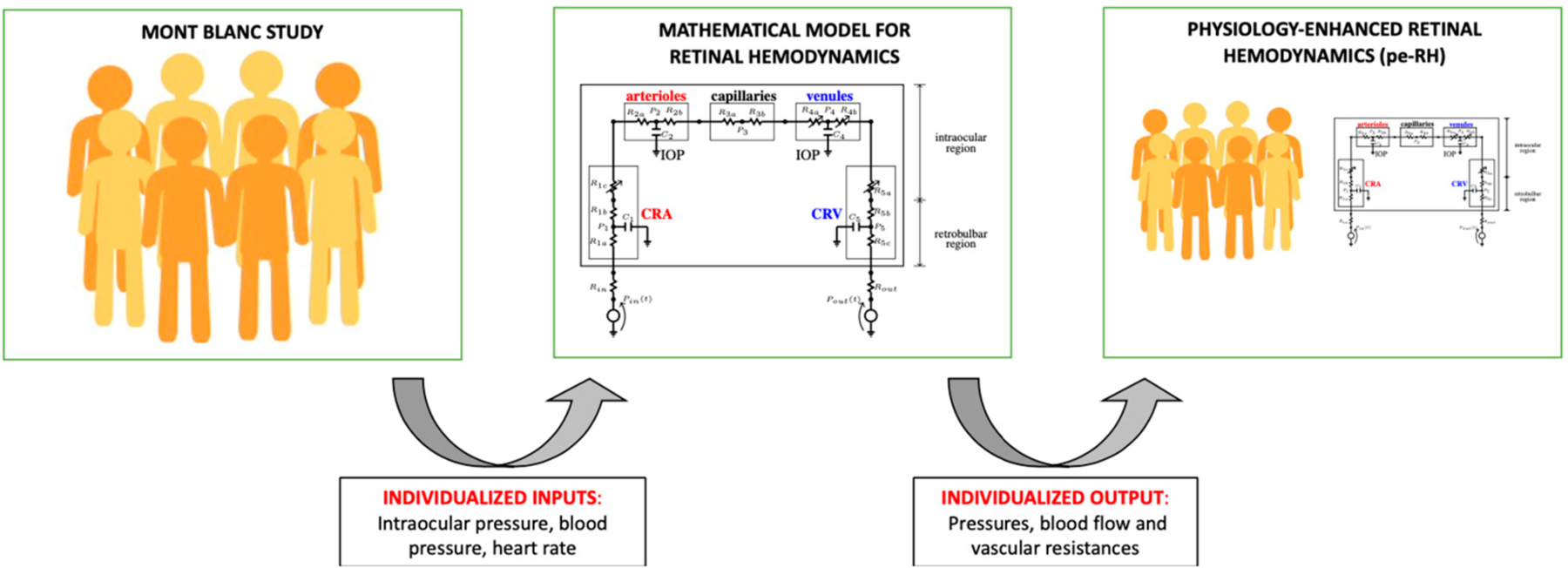
Schematic of the physiology-enhanced retinal hemodynamics (*pe-RH*) analysis. Starting from clinical data (**left**) of intraocular pressure, blood pressure, and heart rate from the Mont Blanc Study, the mathematical model for retinal hemodynamics (**center**) was used to predict pressures, blood flow, and vascular resistances in the retina as individualized outputs, resulting in the *pe-RH* dataset (**right**).

This analysis, henceforth referred to as physiology-enhanced retinal hemodynamics analysis (*pe-RH* analysis), utilized the values of IOP, SBP, DBP, and HR measured on each subject at each altitude as *individualized inputs* for the retinal hemodynamic model. Specifically, SBP, DBP, and HR were combined using formula (3.9) in [41] to define the individualized pressure *P*_*in*_ at the inlet of the retinal vasculature. In the model, the combined action of BP and IOP induces variations in the vascular resistances characterizing the intraocular segments of the CRA (R_1c_), CRV (R_5a_), and the retinal venules (R_4_ = R_4a_ + R_4b_). These variable resistances are indicated by arrows in the mathematical model in the center of Figure 1. We remark that, in this study, we assumed the diameter of arterioles to be constant. The model predicted values of BP, blood flow, and vascular resistances across the retinal vasculature based on individualized inputs yield *individualized outputs* on which statistical analysis can be performed. Results between the two locations were analyzed using a *t*-test with *p* < 0.05 being statistically significant. A schematic illustration of the *pe-RH* analysis is reported in Figure 1.

### 2.3. Mathematical Model of Aqueous Humor Dynamics

The mathematical model proposed and validated in [40] to study aqueous humor (AH) dynamics was also used in combination with the data from the MBS to gain insights into the relationship between altitude and IOP physiology. In the model, IOP is calculated as the result of the balance between AH inflow (*J*_*IN*_) and outflow (*J*_*OUT*_). AH inflow is assumed to be due to (i) active secretion (*J*_*s*_), which is determined by the osmotic pressure difference (OPD), and (ii) ultrafiltration, which is determined by the oncotic pressure difference (*J*_*p*_) and the capillary blood pressure (cBP). AH outflow is assumed to occur through the trabecular meshwork (*J*_*tm*_) and uveoscleral pathways (*J*_*uv*_). In this study, the model was used in a novel way to investigate the physiological mechanisms relating changes in IOP with changes in altitude and BP. Two different analyses were performed, as detailed below.

**Figure 2.**
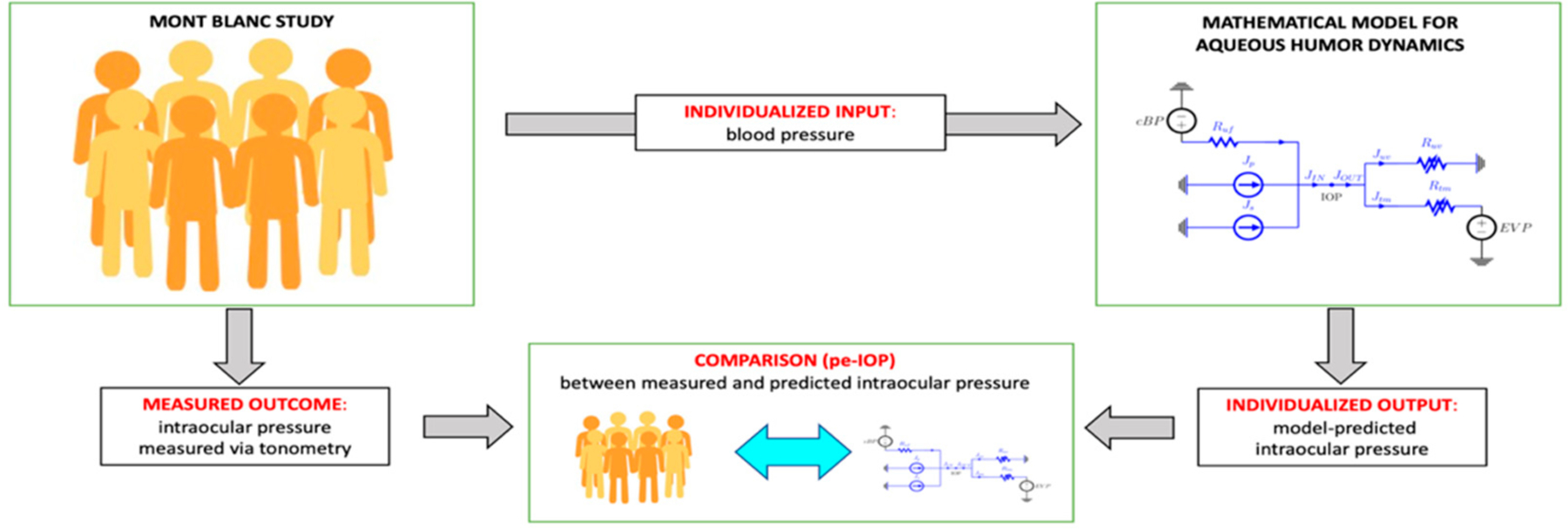
Schematic of the physiology-enhanced intraocular pressure (*pe-IOP*) analysis. Starting from clinical data (**left**) of blood pressure (BP) from the Mont Blanc Study, the mathematical model of aqueous humor dynamics (**right**) was used to predict the intraocular pressure (IOP) value that would correspond to the altitude-related change in BP and then compared to the IOP value measured via tonometry (**center**).

The first analysis, henceforth referred to as physiology-enhanced intraocular pressure analysis (*pe-IOP* analysis), was conducted to test whether the changes in BP associated with changes in altitude can explain the changes in IOP measured via tonometry (Figure 2). For each subject in the study, we computed the mean arterial pressure (MAP) as MAP = 1/3 SBP + 2/3 DBP, and we used it to set the value of cBP as an *individualized input* for the model. Following [39], we set cBP = *a*× MAP, with *a* = 0.29, and we kept other model parameters at their baseline values as listed in [40]. Upon utilizing cBP as an individualized input, the AH model was used to predict the corresponding IOP value as an *individualized output*, which was then compared with the actual IOP value measured on that specific subject at different altitudes (PV, CM, PH).

**Figure 3.**
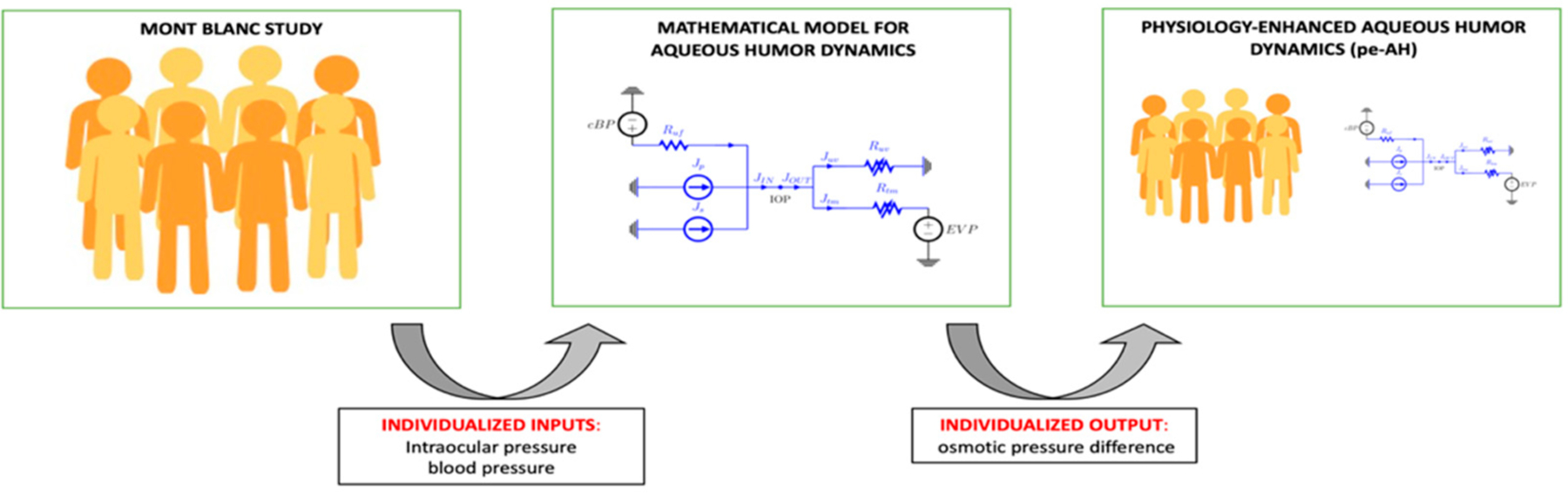
Schematic of the physiology-enhanced aqueous humor (*pe-AH*) analysis. Starting from clinical data (**left**) of intraocular pressure (IOP) and blood pressure (BP) from the Mont Blanc Study, the mathematical model of aqueous humor dynamics (**center**) was used to estimate the value of the osmotic pressure difference (OPD) and enhance the dataset (**right**).

The second analysis, henceforth referred to as physiology-enhanced aqueous humor analysis (*pe-AH* analysis), was conducted to test whether and to what extent changes in OPD may be contributing to IOP changes observed with altitude (Figure 3). For each subject in the study, we used the measured values of MAP and IOP as *individualized inputs*, with cBP scaled from MAP as described above, and we utilized the model to estimate the OPD value that would be necessary to attain a balance between AH inflow and outflow for that specific subject at different altitudes (PV, CM, PH).

## 3. Results

### 3.1. Physiology-Enhanced Data Analytics: Retinal Hemodynamics

**Figure 4.**
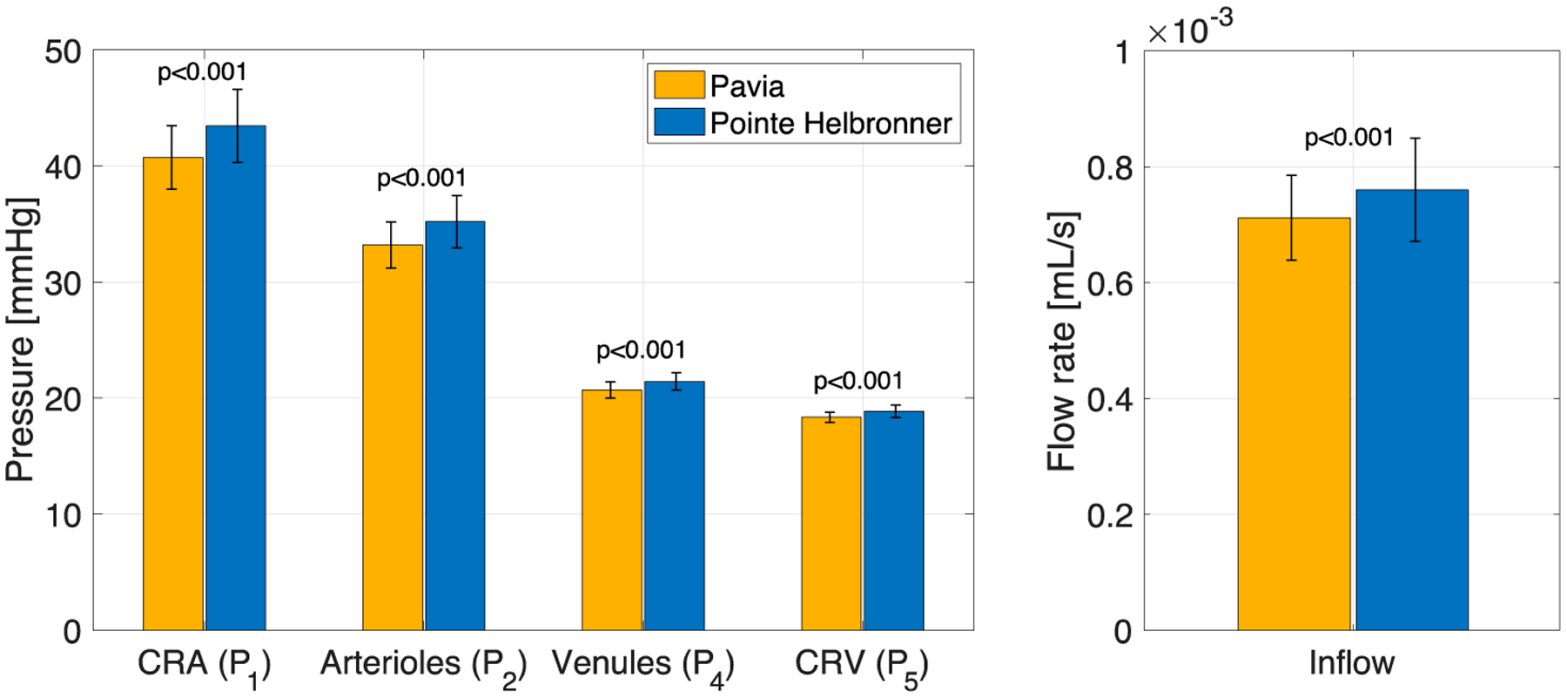
The physiology–enhanced retinal hemodynamics analysis predicted average values and standard deviation of the vascular pressures and blood flow rates in different retinal vascular beds for the two locations/altitudes considered. CRA: central retinal artery; CRV: central retinal vein. Attribution: Carichino L.; Guidoboni G.; Szopos M.; Verticchio Vercellin A.C.; Bruttini C.; Riva, I.; Siesky B.A.; Quaranta L.; Harris A. Effect of altitude on retinal hemodynamics: A physiology-enhanced theoretical analysis. Investigative Ophthalmology & Visual Science June 2021, Vol.62, 560. (Creative Commons Attribution-NonCommercial-NoDerivatives 4.0 International Public License).

**Figure 5.**
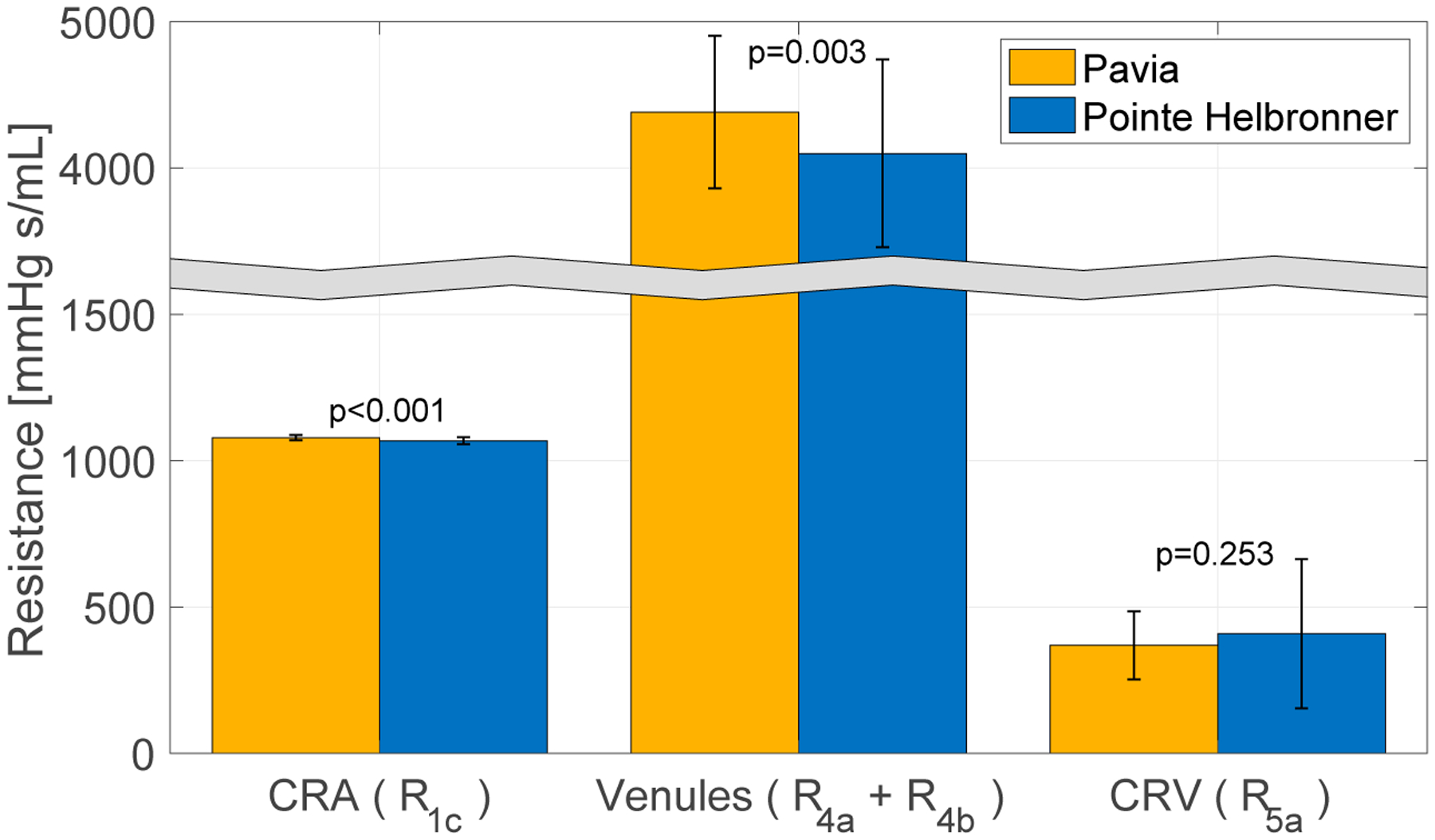
The physiology–enhanced retinal hemodynamics analysis predicted average values and standard deviation of resistances of the intraocular segments of the central retinal artery (CRA) (R_1c_), central retinal vein (CRV) (R_5a_), and retinal venules (R_4_ = R_4a_ + R_4b_) for the two locations/altitudes considered. Attribution: Carichino L.; Guidoboni G.; Szopos M.; Verticchio Vercellin A.C.; Bruttini C.; Riva, I.; Siesky B.A.; Quaranta L.; Harris A. Effect of altitude on retinal hemodynamics: A physiology-enhanced theoretical analysis. Investigative Ophthalmology & Visual Science June 2021, Vol.62, 560. (Creative Commons Attribution-NonCommercial-NoDerivatives 4.0 International Public License).

The results of the pe-RH analysis are reported in Figures 4 and 5. The measured BP values exhibit an increasing trend with altitude, with values of MAP of 83.69 (±6.62) mmHg at PV and 92.82 (±8.43) at PH [32]. Following this increase in systemic BP with altitude, the model predicted blood flow supplying the retina, the model predicted BPs in the CRA, CRV, and retinal arterioles and venules, and the inflow retinal blood flow show a statistically significant increase (*p* < 0.001) with altitude (Figure 4).

The measured IOP values exhibit a decreasing trend with altitude, with values of 11.97 (±2.39) mmHg reported at PV and 11.34 (±2.74) at PH [32]. The *pe-RH* analysis predicted that this decrease in IOP with altitude is linked to a non-monotonic behavior of the retinal vascular resistances shown in Figure 5. In particular, the model shows a statistically significant decrease in the CRA resistance (R_1c_) (*p* < 0.001) and the retinal venules total resistance (R_4_ = R_4a_ + R_4b_) (*p* = 0.003) and a non-significant increase in the CRV resistance (R_5a_) (*p* = 0.253) with altitude. Note that the total resistance in the retinal venules is markedly higher than in the CRA and CRV, suggesting that the venules withstand most of the IOP load on the vasculature.

### 3.2. Physiology-Enhanced Data Analytics: Aqueous Humor Dynamics

**Figure 6.**
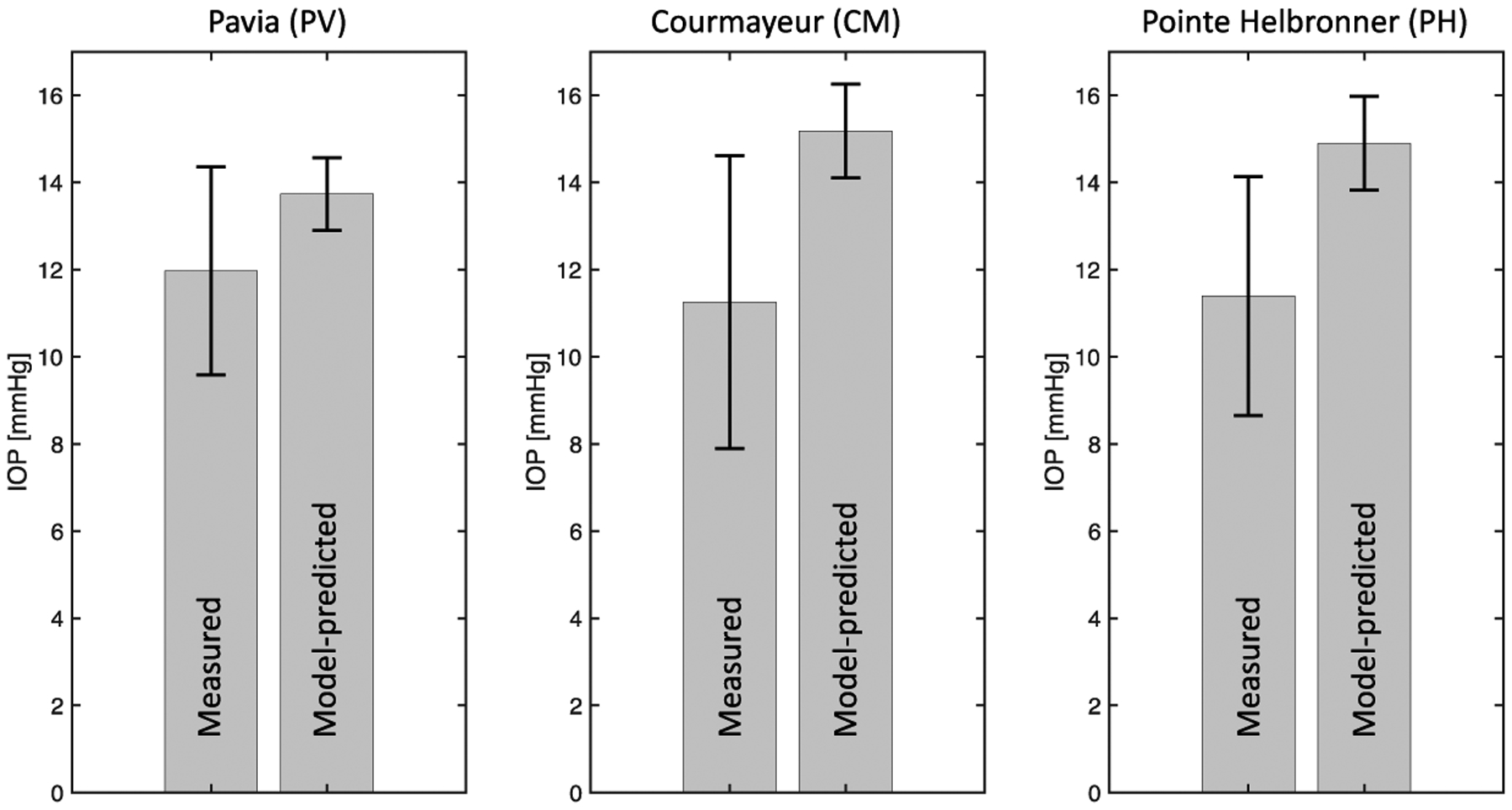
The physiology–enhanced intraocular pressure analysis shows that changes in mean arterial pressure are not sufficient to explain the measured intraocular pressure (IOP) differences at different altitudes, since the measured IOP decreases with altitude while the model predicted IOP increases. Attribution: Guidoboni, G.; Szopos, M.; Verticchio Vercellin, A.C.; Bruttini C.; Riva, I.; Siesky, B.A.; Harris A.; Quaranta L. Physiology-enhanced data analytics to evaluate the effect of altitude on intraocular pressure. Investigative Ophthalmology & Visual Science June 2021, Vol.62, 559 (Creative Commons Attribution-NonCommercial-NoDerivatives 4.0 International Public License).

The results of the *pe-IOP* analysis are reported in Figure 6. As previously indicated, the measured IOP values exhibit a decreasing trend with altitude [32]. The model predicted IOP values exhibit the opposite trend, with mean values of 13.7 mmHg, 15.2 mmHg, and 14.9 mmHg obtained for PV, CM, and PH. This finding shows that changes in MAP are not sufficient to explain the measured IOP differences at different altitudes. As a matter of fact, in this analysis, the MAP value was the only model input to be modified with altitude, thereby assuming that all other factors, including OPD, were given constants that are independent of the subject and the altitude.

**Figure 7.**
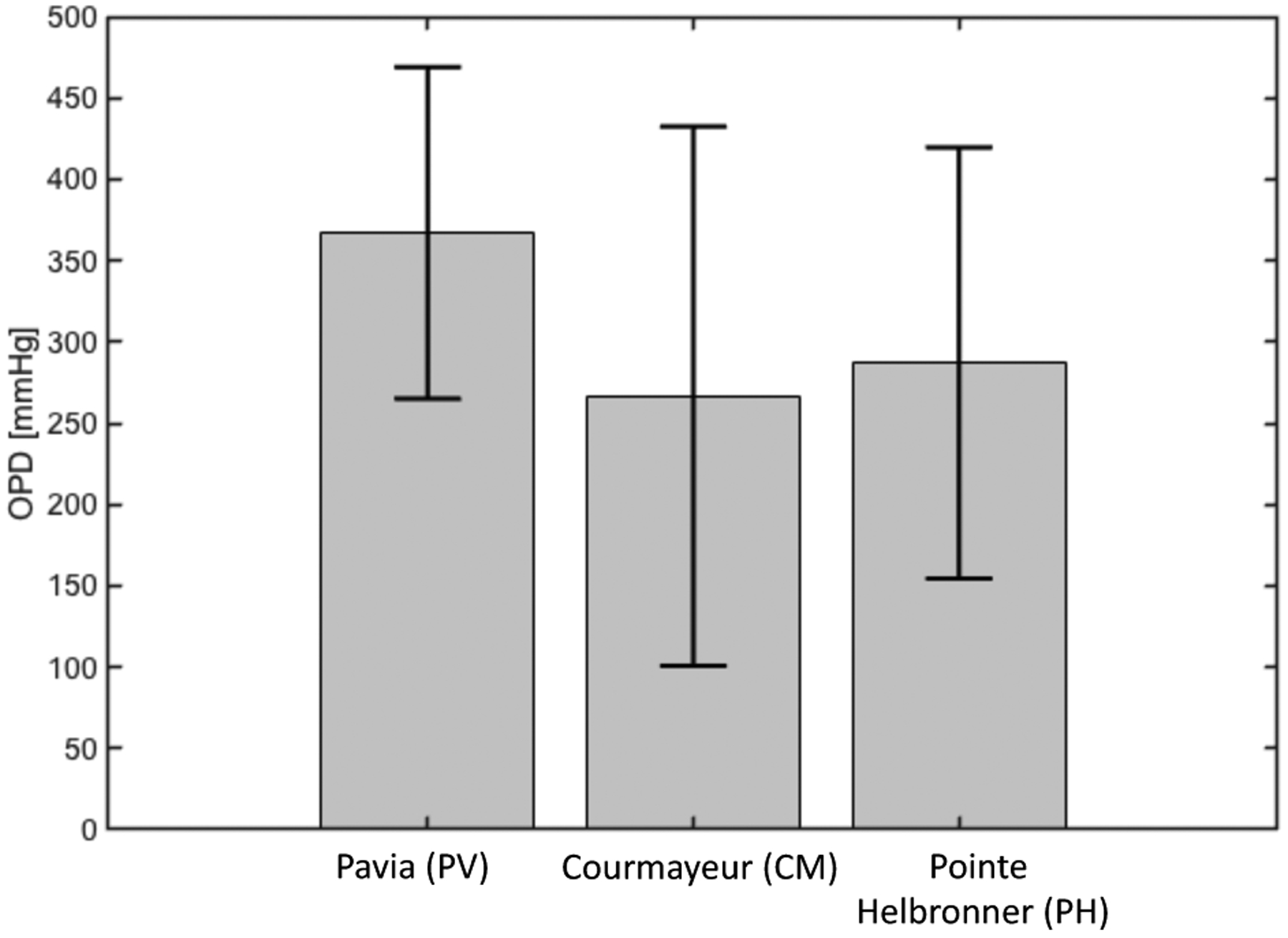
The physiology–enhanced aqueous humor analysis shows that a decrease in osmotic pressure difference (OPD) may underlie the difference in intraocular pressure measured at different altitudes. Attribution: Guidoboni, G.; Szopos, M.; Verticchio Vercellin, A.C.; Bruttini C.; Riva, I.; Siesky, B.A.; Harris A.; Quaranta L. Physiology-enhanced data analytics to evaluate the effect of altitude on intraocular pressure. Investigative Ophthalmology & Visual Science June 2021, Vol.62, 559 (Creative Commons Attribution-NonCommercial-NoDerivatives 4.0 International Public License).

The results of the pe-AH analysis are reported in Figure 7. The results show that changes in OPD, in conjunction with MAP, capture the decreasing trend of IOP with altitude as observed in the MBS. Specifically, MAP values were measured at PV, CM, and PH [32], and the corresponding model predicted values of OPD are 367.0 mmHg, 266.5 mmHg, and 287.0 mmHg.

## 4. Discussion

The relationships between IOP, BP, and ocular hemodynamics during varied elevation are important considerations in the risk management of ophthalmic diseases such as glaucoma, yet they are particularly difficult to investigate in clinical practice. The current literature on altitude and physiologic variables such as IOP, BP, and hemodynamics is particularly difficult to interpret due to the conflicting presence of environmental variables and methodological variances [12–14,23–25]. Specifically, some studies utilized hypobaric chambers to simulate altitude changes, while others followed participants through climbs or lifts to higher elevation. Additionally, each study had wide variation in its measurement timelines and temperature at which the measurements were assessed, which may further influence comparisons. Finally, no specific altitude was consistently utilized across studies, therefore complicating the understanding of what elevation level is required to significantly impact IOP and/or ocular hemodynamics. In order to overcome these limitations, in this study, we used an innovative methodological approach indicated as *physiology-enhanced data analytics* able to integrate experimental data—derived from the MBS [32]—with validated mathematical models [39,40] in order to shed further light on the physiologic relationships between IOP, BP, and ocular hemodynamics with altitude.

First, the mathematical model validated in [39] was here used in combination with the MBS to evaluate how BP and IOP changes at different altitudes may influence retinal hemodynamics. In detail, the *pe-RH* analysis utilized the values of IOP, SBP, DBP, and HR measured on each subject at each altitude in the MBS as *individualized inputs* and provided the values of BP, blood flow, and vascular resistances across the retinal vasculature as *individualized outputs* (Figure 1). With altitude, the experimental data from the MBS showed an increase in BP, which corresponded to model predicted statistically significant increases in the blood flow supplying the retina and BPs in the CRA, CRV, and retinal arterioles and venules (Figure 4). Additionally, the model predicted that the decrease in IOP with altitude found in the MBS was linked, in particular, to a statistically significant decrease with elevation in the total resistance at the level of the retinal venules (Figure 5). Generally, the literature agrees with the findings of this study as several pilot investigations demonstrated short-term increases in retinal blood flow, velocity, and pressures with increasing altitude [8,10,21,33,34]. Our results from the *pe-RH* analysis confirm the data from the literature and also suggest that altitude may significantly increase retinal vessel pressures, alter localized tissue perfusion, and indicate that venules bear the significant portion of the IOP load within the ocular vasculature. This is a novel and important finding that may explain how elevation results in acute increases in retinal perfusion and pressures. In fact, the venous system’s blood vessel walls are less rigid and internally pressurized than on the arterial side, making venous vessels possibly more susceptible to pressure increases from elevation. These results also suggest that the retinal venules may play an important role in the regulation of the blood flow in response to IOP and BP changes with altitude. We recall that, in this study, the mathematical model did not include active regulatory mechanisms that would lead to dilation or constriction of arterioles. Our analysis suggests that such mechanisms may be called into action as a response to changes that are occurring at the level of the venules downstream. Therefore, further studies are needed to investigate the retinal venous compartment as a potential target for future estimation of risk and treatment development in the management of glaucoma.

In addition, we also used the mathematical model validated in [40] on AH dynamics in combination with the experimental data from the MBS to shed further light on the physiological mechanisms between changes in IOP and BP with altitude through two sub-analyses. The *pe-IOP* analysis used the values of MAP derived from the MBS as *individualized inputs* and provided the corresponding IOP values at different altitudes as *individualized outputs*, which were then compared with the actual IOP values collected in the real word (Figure 2). The IOP values predicted by the model exhibited an increasing trend with altitude, while the experimental IOP decreased with elevation, thus showing that changes in MAP are not sufficient to explain the measured IOP differences at different altitudes (Figure 6). Important findings in this context were then derived from the *pe-AH* analysis which used the measured values of MAP and IOP as *individualized inputs* and estimated the OPD values needed to achieve a balance between inflow and outflow of aqueous humor for each subject at different altitudes from the model as *individualized outputs* (Figure 3). Interestingly, the *pe-AH* analysis showed that a decrease in OPD may be the physiologic mechanism underlying the differences in IOP measured at different altitudes, thus suggesting that OPD may play an important role in regulating IOP during changes in altitude. This result is particularly significant because it highlights how mathematical modeling, by estimating parameters that cannot be easily measured experimentally, may help elucidate pathophysiological mechanisms that occur with altitude changes. Further studies will be needed to implement the model to include variables such as temperature and oxygen saturation that may further influence the relationship between altitude, BP, and IOP.

The relationships between altitude, IOP, and hemodynamics are complex, and the current literature does not provide consensus on their associations or possible influence on the risk of glaucoma. Importantly, further research should be specifically designed to account for environmental variables and methodological variances to address the conclusion regarding the association between IOP and altitude. As these relationships are interwoven, ocular hemodynamic variables should also be measured in patients during ascension or ascension-simulated conditions affecting IOP. Mathematical modeling provides an effective tool for enhancing the analysis options of clinical datasets and allows for the testing of hypotheses in a virtual laboratory. These tools are especially important for understanding complex interactions that cannot be viewed in vivo due to anatomical target inaccessibility or imaging and measurement limitations. Identifying and modeling biomarkers such as venous resistance and OPD may provide a framework for understanding altitude’s effects on IOP, ocular hemodynamics, and the risk of ocular pathologies. Additionally, future model analytics may include the effect of temperature and CCT for enhanced specificity and eventually include the effect of altitude on oxygen saturation via autoregulation to better evaluate and understand ocular physiology and potential mechanisms of pathophysiology.

Our study is not without limitations. It is important to acknowledge that while mathematical modeling represents an effective tool for enhancing the analysis options of clinical datasets and allows for the testing of hypotheses in a virtual laboratory, only real-world measurement can provide the final confirmation of the tested hypotheses, and these should be the next steps in confirming our results. Furthermore, the analysis presented in this study does not explicitly include vascular regulatory mechanisms, whose recruitment in response to alterations in the venous side of the circulation remains largely understudied. In addition, the mathematical models presented in our study used data previously collected from the MBS as inputs [32], which presented several limitations as follows: (i) the IOP measurements were performed with tonometers that have not been previously validated to be used at high altitudes; (ii) the influence of acclimatization on the physiologic parameters was not evaluated; and (iii) the study subjects were all healthy subjects with a mean age of 24.8 years. Therefore, further experimental studies are needed to investigate the effect of acclimatization on the physiologic parameters analyzed and should be conducted specifically in older patients with glaucoma as this population may have differing pathophysiological mechanisms that underlie their condition and differentially influence IOP and other physiologic variables at high altitude.

## 5. Conclusions

Our *physiology-enhanced data analytics* methodological approach applied to experimental data derived from the MBS on healthy subjects predicted elevation-induced increases in the blood flow and pressures supplying the retina and a decrease in the total resistance at the level of the retinal venules.

In addition, the model predicted that OPD plays a significant role in IOP regulation at different altitudes, and that high altitude should be considered when assessing IOP and risk in patients with glaucoma. These pilot results need to be confirmed in glaucomatous patients to ensure their applicability in clinical practice. High altitude may potentially represent an environmental factor that could assist in assessing individual risk and/or potentially the manipulation of IOP. Further, these novel experimental data may be used as inputs to mathematical models that can have a crucial role as tools to further the understanding of the mechanisms involved in IOP and ocular hemodynamics. These results are applicable not only in physiologic conditions, as discussed in this study, but also in pathological conditions including glaucoma and other diseases of the retina and optic nerve.
